# Factors Associated with Hepatitis C Antibody Positivity in Korean Adults: A Cross-Sectional Study Based on 2013–2018 Korea National Health and Nutrition Examination Survey

**DOI:** 10.3390/healthcare9101366

**Published:** 2021-10-14

**Authors:** Taehui Kim, Jiyoung Kim

**Affiliations:** 1 Department of Nursing, Joongbu University, 201 Daehak-ro, Geumsan-gun 32713, Chungcheongnam-do, Korea; skyibe@joongbu.ac.kr; 2 Department of Nursing, Chungcheong University, 38 Wolgok-gil, Cheongju-si 28171, Chungcheongbuk-do, Korea

**Keywords:** hepatitis C, adult, infection, prevalence, antibody, public health, prevention, chronic hepatitis

## Abstract

This study aimed to provide basic data on the prevention of hepatitis C infection by identifying factors related to it based on the data from the National Health and Nutrition Examination Survey (KNHANES). The sixth (2013–2015) and seventh (2016–2018) Korean National Health and Nutrition Examination Surveys conducted by the Korean Disease Control and Prevention Agency were analyzed. This is a population-based, nationally representative, multistage, cross-sectional survey of noninstitutionalized persons in Korea. Multivariate regression analysis was used to assess the significance of the variables. A total of 32,942 persons aged >20 years were selected for this study. Among them, 282 tested positive for hepatitis C antibodies, while 32,660 tested negative. Of the 282 persons who tested positive, 48.6% were men and 51.4% were women. The factors associated with hepatitis C infection were age, education level, self-rated health status, and liver cirrhosis. Therefore, there is a need to educate people and implement preventive programs based on age and education levels to reduce the incidence of hepatitis C infections. In addition, it is necessary to include hepatitis C screening as part of the National Health Examination to diagnose hepatitis C infections.

## 1. Introduction

Hepatitis C is one of the most common blood-associated infections. It is estimated that 71 million people worldwide have chronic hepatitis C infection, accounting for 1% of the global population [1]. Hepatitis C virus (HCV) infection may also be acute, and 80% of these cases progress into chronic hepatitis [2]. Additionally, chronic hepatitis patients undergo disease progression and develop cirrhosis, liver fibrosis, and hepatocellular carcinoma, which eventually lead to death [2,3].

North America, north and west European countries, Australia, and Japan have the lowest prevalence of HCV infections (less than 2%), while Africa and Middle Eastern countries have a prevalence of over 10% [4]. The number of HCV infected persons in China (29.8 million), Egypt (11.8 million), India (18.2 million), and Pakistan (9.4 million) is high, although the prevalence is low or moderate because of the large population of these countries [4].

The cause of HCV infection varies from country to country, though it is largely related to the transfusion of blood or blood products, hemodialysis, use of intravenous drugs, and organ transplantation [5]. The World Health Organization (WHO) considers unsafe medical practices and drug abuse as new causes of HCV infection [1]. A risk factor for HCV infection in the United States and Australia is intravenous drug abuse, which in Japan is highly correlated with age, where the prevalence of HCV infection is higher in people aged over 55 years [4,6,7].

It is estimated that approximately 290,000 Korean adults are positive for HCV antibodies. As age increases, a higher prevalence has been reported among women than men [3]. The prevalence of HCV infection was found to be 48.4–79.2% among intravenous drug users, 5.9–14.7% among persons with chronic kidney disease, and 5.0–6.3% among those with human immunodeficiency virus (HIV) infections [7]. Previous study reports have shown that in Korea, the risk factors for HCV infection include needle cuts, dental treatment, tattoos, transfusion requirements before the age of 20, and multiple sexual partners [8,9]. In another study, injectable hallucinogens, a history of acute hepatitis, a drinking history, tattoos, and moxibustion were reported as causes of HCV infection; the use of injectable hallucinogens was determined as the most correlated risk factor for HCV infection in Korea [10]. Previously, several infections were associated with blood transfusion and dental treatment [3]. With HCV screening tests for those undergoing blood transfusions and improved dental infection control, the occurrence of associated infections decreased [3] in hepatitis C patients with chronic kidney diseases, and those patients coinfected with HIV or hepatitis B virus (HBV) [7]. Public awareness regarding hepatitis C improved from 10% in 2009 to 34.0% in 2019, although this is lower than that for hepatitis B (79.3%) and hepatitis A (72.8%) [11]. In order to include hepatitis C in the National Health Examination, the Korean Disease Control and Prevention Agency (KDCA) provided free examinations from 1 September to 31 October 2020 for adults born after 1964. Legislative procedures to include hepatitis C in the National Health Examination are also being discussed in the National Assembly [12]. Additionally, unlike hepatitis A and B, hepatitis C does not have a preventive vaccine; however, it can be cured by treatment for approximately three months if detected early [3]. These changes in the medical and social environment along with improvement in public awareness regarding HCV infections might have resulted in changes to the risk factors associated with it. Therefore, the current factors related to HCV infection are considered different from those described previously, and research is thus required to look into this aspect.

As such, this study aimed to identify factors related to HCV infection based on the data collected from the National Health and Nutrition Examination Survey (KNHANES) conducted by the KDCA and recommend preventive measures.

## 2. Methods

### 2.1. Subjects

This study analyzed data from the sixth (2013–2015) and seventh (2016–2018) KNHANES conducted by the KDCA. The KNHANES is a nationally representative, population-based, multistage, cross-sectional survey of noninstitutionalized persons in Korea that is conducted annually and divided into three years. This is a cross-sectional study that analyzed factors related to HCV infection in Korean adults. The surveys were conducted using a stratified, multistage, clustered probability sampling method based on geographical area, age, and gender so that the results would be representative of the entire Korean population in a year. For this study, 32,942 individuals over the age of 20 were selected (5127 in 2013, 4949 in 2014, 5185 in 2015, 5828 in 2016, 5871 in 2017, and 5985 in 2018). Among them, 282 tested positive for hepatitis C antibodies and 32,660 tested negative. Subjects who tested positive for hepatitis C antibodies were considered hepatitis C patients.

The sixth and seventh KNHANES were conducted with the approval of the Research Ethics Review Committee of the KDCA. The KNHANES data are open for use by scientists through a simple procedure. Further, the KDCA complies with the Personal Information Protection Act and the Statistics Act and provides only data that has been de-identified so that the privacy of individuals is protected. Therefore, approval from the institutional ethics committee was not required.

### 2.2. Variables

#### 2.2.1. Dependent Variable

HCV infection was defined as the presence of hepatitis C antibodies detected through a liver function test. The value for a positive test result was considered 1 and that for a negative one was considered 0.

#### 2.2.2. Independent Variable

The demographic and social characteristics included gender, age, household income quartile, education level, and working status. The prevalence of HCV infection increased with age; therefore, the study subjects were categorized as those in their 20s, 30s, 40s, 50s, and ≥60s. Based on their education level, the subjects were classified into the following groups: below elementary school (a private school for the study of Chinese classics or never attended school or elementary school), middle school, high school, and college or higher. According to their working status, the subjects were classified into two groups: ‘yes’ and ‘no’.

Health, behavior, and disease-related characteristics included stress perception, self-rated health status, current smoking status, smoking history, alcohol consumption, binge drinking frequency, and body mass index (BMI). The following diseases were included: cerebrovascular accident (CVA), myocardial infarction (MI) or angina, liver cancer, cirrhosis, hepatitis B, diabetes mellitus (DM), and kidney failure. Previous studies have shown that one of the risk factors for hepatitis C is hemodialysis [5]. Further, chronic hepatitis C is associated with insulin resistance and cardiovascular disease [13]. Therefore, cardiovascular diseases and diseases related to vascular procedures were included in this analysis. Based on the self-rated health status, the subjects were classified into good (good or very good), moderate, and poor (poor or very poor) groups. The categories based on the current smoking status were non-smoker, ex-smoker (smoked in the past, but not currently), and smoker. According to the smoking history, the subjects were classified into the following groups: less than 1 year, 1 to less than 10 years, and 10 years or more. Considering the amount of alcohol consumed at a time, the groups were low-risk drinking (1–6 cups) and high-risk drinking (7 cups or more). The categories based on the frequency of binge drinking were nothing, less than once a month, about once a month, about once a week, and almost every day. Based on BMI, the subjects were divided into underweight (<18.5 kg/m^2^), normal (18.5–24.9 kg/m^2^), and obese (≥25 kg/m^2^) groups.

### 2.3. Statistical Analysis

For all analyses, sample weights were included, and the weighted frequencies were computed. The differences in these variables between subjects with and without hepatitis C antibodies were compared using a chi-square (χ^2^) test. The factors related to a positive hepatitis C antibody test in the subjects were analyzed by logistic regression analysis. Logistic regression analysis included only statistically significant variables (*p* < 0.05) determined from the χ^2^ test. All analyses were carried out using SPSS software (version 24.0; IBM, Armonk, NY, USA) (complex samples).

## 3. Results

Table 1 shows the difference in the sociodemographic characteristics between the two groups based on presence or absence of hepatitis C antibodies. There were 32,942 subjects over 20 years, of which 18,492 were women and 14,450 were men. Among them, 282 were positive for hepatitis C antibodies (121 men and 161 women). Although more women than men were positive for hepatitis C antibodies, the difference was not statistically significant (χ^2^ = 0.287, *p* = 0.505). The hepatitis C antibody positivity rate was 0.86%, and it increased with age; 43.8% of them were over 60 years of age, and there was a significant difference between the hepatitis C antibody-positive and -negative groups (χ^2^ = 97.437, *p* < 0.001). In terms of household income, the middle-income group exhibited the highest percentage (53.9%) of hepatitis C antibody positivity. People who were positive for hepatitis C antibodies (29.3%) had a lower income than those who were negative (14.9%). Considering education level, the number of hepatitis C antibody-negative subjects was the highest (40.1%) among people with a college level or higher education, while the highest number of hepatitis C antibody-positive subjects (34.9%) had an elementary school and below education. There were significant differences between the two groups in terms of household income (χ^2^ = 34.887, *p* < 0.001), education (χ^2^ = 71.178, *p* < 0.001), and working status (χ^2^ = 10.969, *p* < 0.001).

The difference between the health, behavior, and disease characteristics among the hepatitis C antibody-positive and negative groups are shown in Table 2. There were significant differences between the two groups in terms of self-rated health status (χ^2^ = 41.730, *p* < 0.001), smoking history (χ^2^ = 4.732, *p* = 0.010), diagnosis of CVA (χ^2^ = 11.436, *p* < 0.001), MI or angina (χ^2^ = 0.089, *p* = 0.417), liver cancer (χ^2^ = 3.862, *p* < 0.001), liver cirrhosis (χ^2^ = 32.665, *p* < 0.001), hepatitis B (χ^2^ = 1.795, *p* = 0.038), and DM (χ^2^ = 3.911, *p* = 0.010).

Logistic regression analysis was performed with the statistically significant variables obtained from the χ^2^ test (Table 3), and it revealed that the factors related to hepatitis C positivity were age, education, self-rated health status, and liver cirrhosis. The odds ratio (OR) for hepatitis C antibody positivity increased with age; the OR for older adults aged 60 years and above was 3.331 (95% confidence interval [CI]: 1.853–5.987). Furthermore, the risk of hepatitis C antibody positivity decreased with higher levels of education. Therefore, the OR of those with an elementary school or lower education was 1, while that of those with college level or higher education status was 0.592 (95% CI: 0.410–0.854). Among health, behavior, and disease characteristics, self-rated health status showed a statistically significant result (*p* < 0.001), with the OR decreasing as health status improved. Considering disease diagnosis, people with liver cirrhosis were found to be 8.661 times more likely to have hepatitis C antibody positivity than those without it (95% CI: 4.641–16.164). In contrast, liver cancer and hepatitis B diagnoses were not associated with a positive hepatitis C antibody test.

## 4. Discussion

The purpose of this study was to identify factors related to the prevalence of HCV infections using the raw data from the sixth and seventh KNHANES. Among the 32,942 adults included in this study, 282 were positive for hepatitis C antibodies, with a positivity rate of 0.86%. This is lower than the value of 1.5% that was seen in an earlier study of hepatitis C antibody prevalence in subjects 18 years or older; however, it is similar to the value of 0.9%, which was the result of a study conducted on the prevalence of hepatitis C ribonucleic acid (RNA) [4,6]. Therefore, the prevalence of hepatitis C in Korea was comparable to that in the United States, Canada, Iran, and France [4,6,14,15]. In this study, the prevalence of hepatitis C antibodies was higher in women than in men and increased with age. These results are similar to those of previous epidemiological investigations [3,10]. In another study, the prevalence of hepatitis C increased among those over 55 years of age; this was a finding similar to ours [16]. Previous study reports have shown that lower socioeconomic status (education, income, and job status) is correlated with higher prevalence of hepatitis C [15,17]. The results of this study showed that there were differences between the hepatitis C positive and negative groups in terms of income, education, and working status, although, only education status was analyzed as a related factor in the logistic regression analysis. In addition, hepatitis C antibody positivity according to education level and self-rated health status decreased with a higher education level and better self-rated health status. This supports research findings that people with higher education and income are healthier. Furthermore, education and income status interact with health and affect health indicators such as blood pressure [18]. It is widely known that smoking is harmful to health and increases susceptibility to infections, such as bacteremia and pneumonia [19,20]. A study by Chae showed that the smoking status was a risk factor for HCV infection [10]. Although there was a difference between the two groups regarding smoking history, the logistic regression analysis revealed that the difference was not significant. However, it was difficult to find the results of this research on the epidemiological relationship between smoking and hepatitis C infection. Nevertheless, HCV infection in patients who smoke is more likely to progress to liver fibrosis or cancer [4]. Further, smoking affected survival after surgery in cases where cancer resulted from HCV infection [21,22]. In this study, 26.3% of HCV antibody-positive individuals were smokers, which implies that educating people on smoking cessation is required. The research by Chae also showed that drinking history was associated with HCV infection, which is a finding that is different from that of this study [10]. This may be because of the difference in the time of the variable measurement. In view of the physiological properties of alcohol, which is decomposed in the liver, drinking history might have affected the current hepatitis C infection of the subjects [23]. However, alcohol consumption in this study indicates the relationship between alcohol consumption and hepatitis C infection is unclear. 

People infected with HCV have a higher prevalence of comorbidities, such as CVA, compared with the general population [24,25]. Our study showed similar results, although they were not statistically significant (OR, 1.493). In the past, the same hemodialysis machines were used for people with infectious diseases and those without them, resulting in many cases of cross infection. Furthermore, HCV infections can occur in patients with angina or MI and renal failure as a health-associated infection because of frequent hospital admissions and procedures. However, this study does not demonstrate any significant relationship between these diseases and HCV infection. These results suggest that the management of health-associated infections in Korea is effective. In 2010, the Korean government initiated the healthcare accreditation system to emphasize patient safety and quality of care. This has resulted in improved infection control and management, which is directly linked to patient safety. Before the introduction of this system, these preventive measures were poorly implemented by hospitals owing to the associated cost.

Our study has a few limitations. We omitted variables such as tattoos, intravenous drug use, and HIV infections, which were risk factors considered in previous studies. Additionally, the findings of this study were not significantly different from those of previous studies. Despite this, our study reveals the differences between the past and present risk factors associated with HCV infections. Moreover, government policies related to public health are significant and can either improve or worsen it. Therefore, the diagnosis of HCV infections through the National Health Examination could help in reducing the associated morbidity and enable people to enjoy a healthier life.

## 5. Conclusions

This study showed that there were differences between the HCV antibody positive and negative groups in terms of age, household income, education, working status, self-rated health, smoking history, and diagnosis of CVA, liver cancer, liver cirrhosis, and diabetes. The final regression analysis demonstrated that age, education, self-rated health status, and liver cirrhosis were factors associated with HCV infection. These results suggest that age-specific education regarding HCV infection is required, especially concerning the risk of progression to liver cirrhosis. In addition, it is necessary to include hepatitis C as part of the National Health Examination to diagnose HCV infections.

## Figures and Tables

**Table 1:** Differences in HCV antibody status of Korean adults by socio-demographic characteristics.

Variables	Categories	Total (*n* = 32,942)	HCV Antibody Positive		χ^2^ (*p*)
			No (*n* = 32,660)	Yes (*n* = 282)	
		n * (%) ^ †^	n * (%) ^ †^	n * (%) ^ †^	
Gender	Male	14,450 (50.3)	14,329 (50.3)	121 (48.6)	0.287 (0.505)
	Female	18,492 (49.7)	18,331 (49.7)	161 (51.4)	
Age	20s	3653 (16.9)	3644 (17.0)	9 (5.4)	97.437 (*p* < 0.001)
	30s	5412 (19.1)	5399 (19.1)	13 (6.7)	
	40s	6267 (21.4)	6234 (21.4)	33 (16.5)	
	50s	6578 (20.3)	6516 (20.3)	62 (27.6)	
	≥60s	11,032 (22.3)	10,867 (22.1)	165 (43.8)	
House income	Low	6118 (15.0)	6026 (14.9)	92 (29.3)	34.887 (*p* < 0.001)
	Middle	17,167 (53.9)	17,027 (54.0)	140 (49.5)	
	High	9497 (31.1)	9448 (31.2)	49 (21.1)	
Education	≤Elementary	6601 (15.2)	6492 (15.0)	109 (34.9)	71.178 (*p* < 0.001)
	Middle	3215 (8.9)	3175 (8.9)	40 (14.3)	
	High school	10,074 (36.0)	10,009 (36.0)	65 (29.5)	
	≥College	11,013 (39.9)	10,968 (40.1)	45 (21.4)	
Working status	No	12,066 (35.1)	11,932 (35.0)	134 (46.1)	10.969 (*p* < 0.001)
	Yes	18,803 (64.9)	18,678 (65.0)	125 (53.9)	

* n is non-weighted value; ^†^ % is weighted value to correct for the target popluation.

**Table 2:** Differences in HCV antibody status of Korean adults by health behavior and disease characteristics.

Variables	Categories	Total (*n* = 32,942)	HCV Antibody Positive		
			No (*n* = 32,660)	Yes (*n* = 282)	χ^2^ (*p*)
		n * (%) ^ †^	n * (%) ^ †^	n * (%) ^ †^	
Stress perception	Low feeling	23,711 (72.7)	23,509 (72.7)	202 (74.0)	0.201 (0.574)
	High feeling	8240 (27.3)	8170 (27.3)	70 (26.0)	
Self-rated health status	Bad	5971 (17.3)	5878 (17.2)	93 (33.8)	41.730 (*p* < 0.001)
	Moderate	16,095 (51.8)	15,968 (51.9)	127 (48.6)	
	Good	9009 (30.8)	8967 (30.9)	42 (17.6)	
Smoking status	Non smoker	19,189 (56.1)	19,038 (56.2)	151 (52.0)	1.923 (0.287)
	Ex-smoker	6714 (21.1)	6646 (21.1)	68 (21.8)	
	smoker	6054 (22.8)	6002 (22.8)	52 (26.3)	
Past smoking period(years)	<1	5517 (16.3)	5456 (16.3)	61 (19.5)	4.732 (0.010)
	1–10	1096 (4.0)	1089 (4.0)	7 (1.9)	
	≥10	25,932 (79.7)	25,722 (79.7)	210 (78.6)	
Alcohol consumption	Low risk drinking	17,281 (69.9)	17,157 (69.9)	124 (75.6)	2.251 (0.079)
	High risk drinking	5919 (30.1)	5883 (30.1)	36 (24.4)	
Binge drinking	None	9195 (34.3)	9133 (34.3)	62 (32.1)	1.291 (0.786)
	<1/month	4612 (20.9)	4577 (20.9)	35 (23.3)	
	1/month	3659 (17.7)	3636 (17.7)	23 (18.5)	
	1/week	4182 (19.9)	4156 (19.9)	26 (17.7)	
	Almost everyday	1542 (7.3)	1528 (7.3)	14 (8.5)	
BMI	Low	1221 (4.1)	1212 (4.1)	9 (3.1)	1.913 (0.245)
	Normal	20,374 (61.6)	20,206 (61.7)	168 (58.7)	
	Obesity	11,273 (34.3)	11,169 (34.3)	104 (38.2)	
CVA dx.	No	30,345 (98.3)	30,097 (98.3)	248 (94.5)	11.436 (*p* < 0.001)
	Yes	726 (1.7)	712 (1.7)	14 (5.5)	
MI or Angina dx.	No	30,799 (99.3)	29,948 (98.0)	254 (98.3)	0.089 (0.417)
	Yes	262 (0.7)	851 (2.0)	8 (1.7)	
Liver cancer dx.	No	31,013 (99.7)	30,755 (99.7)	258 (95.8)	3.862 (*p* < 0.001)
	Yes	40 (0.3)	94 (0.3)	3 (4.2)	
Cirrhosis dx.	No	30,943 (99.7)	30,694 (99.7)	249 (95.4)	32.665 (*p* < 0.001)
	Yes	106 (0.3)	94 (0.3)	12 (4.6)	
Hepatitis B dx.	No	30,636 (98.7)	30,383 (98.7)	253 (97.6)	1.795 (0.038)
	Yes	413 (1.3)	405 (1.3)	8 (2.4)	
DM dx.	No	28,950 (92.8)	28,717 (92.8)	233 (89.2)	3.911 (0.010)
	Yes	2948 (7.2)	2909 (7.2)	39 (10.8)	
Renal failure dx.	No	30,946 (99.7)	30,688 (99.7)	258 (99.2)	1.329 (0.074)
	Yes	103 (0.3)	100 (0.3)	3 (0.8)	

* n is non-weighted value; ^†^ % is weighted value to correct for the target popluation.

**Table 3:** Associated factors with positive HCV antibody status (*N* = 32,942).

Characteristics	Variables	Categories	HCV Antibody Positive		
			OR	95% CI	*p*
Socio- Demographic characteristics	Age	20s (Ref.)	1		<0.001
		30s	0.812	0.387–1.704	
		40s	2.189	1.179–4.066	
		50s	3.159	1.752–5.694	
		≥60s	3.331	1.853–5.987	
	House income	Low (Ref.)	1		0.294
		Middle	0.840	0.648–1.090	
		High	0.767	0.533–1.103	
	Education	≤Elementary (Ref.)	1		0.030
		Middle	0.826	0.584–1.169	
		High school	0.687	0.496–0.952	
		≥College	0.592	0.410–0.854	
	Working status	No (Ref.)	1		0.346
		Yes	0.901	0.726–1.119	
Health behavior and disease characteristics	Self-rated health status	Bad (Ref.)	1		<0.001
		Moderate	0.654	0.507–0.843	
		Good	0.479	0.343–0.670	
	Past smoking period (years)	<1 (Ref.)	1		0.433
		1–10	0.626	0.307–1.275	
		≥10	0.924	0.704–1.212	
	CVA dx.	No (Ref.)	1		0.071
		Yes	1.493	0.966–2.308	
	Liver cancer dx.	No (Ref.)	1		0.434
		Yes	1.474	0.557–3.905	
	Cirrhosis dx.	No (Ref.)	1		<0.001
		Yes	8.661	4.641–16.164	
	Hepatitis B dx.	No (Ref.)	1		0.864
		Yes	0.934	0.428–2.040	
	DM dx.	No (Ref.)	1		0.051
		Yes	0.704	0.495–1.002	

## Data Availability

The datasets was analyzed from ‘https://knhanes.kdca.go.kr/knhanes/sub03/sub03_02_05.do (accessed on 13 October 2021)’.
